# A Mission Planning Approach for Precision Farming Systems Based on Multi-Objective Optimization

**DOI:** 10.3390/s18061795

**Published:** 2018-06-02

**Authors:** Zhaoyu Zhai, José-Fernán Martínez Ortega, Néstor Lucas Martínez, Jesús Rodríguez-Molina

**Affiliations:** Departamento de Ingeniería Telemática y Electrónica (DTE), Escuela Técnica Superior de Ingeniería y Sistemas de Telecomunicación (ETSIST), Universidad Politécnica de Madrid (UPM), C/Nikola Tesla, s/n, 28031 Madrid, Spain; jf.martinez@upm.es (J.-F.M.O.); nestor.lucas@upm.es (N.L.M.); jesus.rodriguezm@upm.es (J.R.-M.)

**Keywords:** precision farming system, multi-agent system, agent coalition, multi-objective optimization, mission planning approach

## Abstract

As the demand for food grows continuously, intelligent agriculture has drawn much attention due to its capability of producing great quantities of food efficiently. The main purpose of intelligent agriculture is to plan agricultural missions properly and use limited resources reasonably with minor human intervention. This paper proposes a Precision Farming System (PFS) as a Multi-Agent System (MAS). Components of PFS are treated as agents with different functionalities. These agents could form several coalitions to complete the complex agricultural missions cooperatively. In PFS, mission planning should consider several criteria, like expected benefit, energy consumption or equipment loss. Hence, mission planning could be treated as a Multi-objective Optimization Problem (MOP). In order to solve MOP, an improved algorithm, MP-PSOGA, is proposed, taking advantages of the Genetic Algorithms and Particle Swarm Optimization. A simulation, called precise pesticide spraying mission, is performed to verify the feasibility of the proposed approach. Simulation results illustrate that the proposed approach works properly. This approach enables the PFS to plan missions and allocate scarce resources efficiently. The theoretical analysis and simulation is a good foundation for the future study. Once the proposed approach is applied to a real scenario, it is expected to bring significant economic improvement.

## 1. Introduction

World population as of 2018 has become more than 7 billion and is estimated to reach 11.2 billion by 2100. Such a high population requires large quantities of food for survival. However, conventional agriculture is unlikely to fulfill the demand for food in the future [1], because it will requires massive human labor and more time to produce the necessary amount of food. Furthermore, conventional agriculture wastes too much resources, such as water and pesticides, due to their imprecise use. Under this circumstance, intelligent agriculture is put forward to improve the traditional one [2,3,4]. The concept “precision farming” as a branch of intelligent agriculture has drawn much attention recently. A Precision Farming System (PFS), on the basis of “4S” (RS, GIS, DSS and GPS), has the capability of regularly performing a set of agricultural operations in a specific area at a specific time [5]. Generally, the precision farming system obtains the environmental data through Remote Sensing (RS) [6,7] to monitor the nutritional status of crops and moisture of soil. Then, the PFS analyses the obtained information by the Geographic Information System (GIS) [8]. Afterwards, the PFS relies on the Decision Support System (DSS) to make a detailed operation plan to distribute agricultural missions [9,10,11]. Lastly, the PFS uses the Global Positioning System (GPS) to guide the agricultural machinery to perform precise operations [12,13]. With the help of PFS, large quantities of food may be produced in a shorter period. Also, limited resources can be allocated in a more reasonable way [14].

Research activities on intelligent agriculture have been continuously conducted in these two last decades. Modern science has been introduced to improve the agricultural approaches [15,16,17,18,19]. Researchers especially focus on the domains of monitoring, control, logistics and prediction. Monitoring objectives include air condition [20], soil [21], water [22], plant [23] and livestock [24]. Control objectives include fertilizer and pesticide [25], illumination [26] and access [27]. Meanwhile, the agricultural logistics is improved by introducing technologies, like Wireless Sensor Networks (WSN) and Radio Frequency Identification (RFID) [28]. Forecasting of agricultural products and farming lands are also taken into account [29].

In PFS, the decision support system plays a significant role, because it is responsible for the mission planning process. Mission planning is an organization process of defining the operation strategy and making decisions on placing a certain amount of resources to complete agricultural missions. It may also refer to a control mechanism for guiding the implementation orders [30]. When multiple machineries or robots work cooperatively in order to complete a complex mission, the mission planning problem must always be considered. Since a mission is usually too difficult for a single machine to complete it, then the mission has to be divided and assigned to certain pieces of equipment. Consequently, a feasible mission plan must be generated. Branko et al. [31] came up with the idea to generate mission plans for underwater robotics by using a genetic planner. Their research is done in correlation with the Smart and Networking Underwater Robots in Cooperation Meshes (SWARMs) project and focuses on the underwater mission planning for different robots, like Unmanned Aerial Vehicles (UAVs) and Remotely Operated Vehicles (ROVs). As stated in their paper, though the genetic planner is designed for underwater missions, the planning approach is universal and not domain dependent. Inspired and learned from the contributions of SWARMs project, the mission planning approach for underwater environment could also be applicable to intelligent agriculture. Since various agricultural machineries are deployed in the farming fields, how to coordinate them and generate feasible plans for agricultural missions urgently needs to be solved. For agricultural applications, UAVs are usually involved in monitoring purposes, or refitted as flying pesticide sprayers. In this paper, we have studied how to coordinate multiple UAVs to complete agricultural missions. For an agricultural mission, there are several feasible planning strategies. However, each strategy may achieve different economy benefits and consume different resources. Ma [32] studied the dynamic task allocation and presented a simple example of mission planning, illustrating that expected benefit and consumed resources may vary from different mission planning strategies. Generally, the mission planning strategies for agricultural missions should consider two main objectives: expected benefit and cost [33].

The sub-objectives of the expected benefit include:

- *Efficiency*: This sub-objective indicates that UAVs could complete the assigned mission within a given time interval.
- *Probability of handling with the mission*: This sub-objective indicates that a UAV’s capability of completing the assigned mission.
- *Economy benefit*: This sub-objective indicates the obtained benefit after completing the assigned mission.

Meanwhile, the sub-objectives of cost include:

- *Energy consumption*: During the mission execution, UAVs have to consume fuel, battery or any other energy resources to perform operations. This sub-objective indicates the consumption rate for UAVs per hour.
- *Equipment loss*: During the mission execution, UAVs definitely cause equipment loss due to mechanical wear; tire and engine wear of the vehicles must be especially taken into account. This sub-objective indicates the loss rate per hour.
- *Malfunction risk*: During the mission execution, UAVs may suffer from mechanical malfunction, especially for the old UAVs. Meanwhile, UAVs may perform operations under bad environment. For example, UAVs are more likely to crash during rainy days. Hence, this sub-objective indicates the risk level for the UAVs

Therefore, the agricultural mission planning could be regarded as a Multi-objective Optimization Problem (MOP), also known as multi-objective programming problem. The MOP is concerned with mathematical computations and studies the approach to optimize multiple objective functions simultaneously in a given time interval, in order to fulfill the requirement of several criteria. Usually, the objective functions are in conflict with each other and have their influence on certain criteria. It is impossible to find a solution to achieve the best performance of each objective function at the same time. In the precision farming system, the optimal mission planning strategy should achieve the balance of benefit and cost. 

Research works on multi-objective optimization problem in intelligent agriculture are mainly focused on constructing agricultural models and developing optimization algorithms [34,35]. Groot et al. proposed the FarmDESIGN model, a bio-economical farm model to evaluate the productive, economic and environmental farm performance. A large set of Pareto optimal alternative farm configurations are generated by running a multi-objective optimization algorithm [36]. Cardoso et al. proposed a multi-objective numerical solution in biological pest control for soybean crops. Their proposal took into consideration the cost of application of the control action and the cost of economic damage. To solve the multi-objective optimization problem, an evolutionary algorithm, called NSGA-II, was introduced [37]. Galan-Martin et al. presented a novel systematic method for agriculture planning to allocate rain fed and irrigated cropping areas. This method is able to enhance food availability and reduce the environmental impact of agricultural. The allocation problem is regarded as a multi-objective optimization problem. The Pareto optimal solution set is generated by running optimization algorithm [38]. 

In this paper, a precision farming system is proposed and treated as a Multi-Agent System (MAS) with an improved federal architecture. Within the PFS, multiple UAVs are treated as autonomous agents and supposed to complete the agricultural missions cooperatively. Meanwhile, the mission planning problem in the PFS is considered as a multi-objective optimization problem. Agent candidates who fulfill the mission requirement will then negotiate with the control terminal through a mission auction. The biddings from agent candidates are calculated by an improved optimization algorithm, MP-PSOGA. This improved algorithm takes advantages of the Particle Swarm Optimization and Genetic Algorithms. The output of MP-PSOGA is the Pareto optimal solution set, representing the optimal mission planning strategies for the established agricultural missions.

To implement the precision farming system and mission planning approach, the main objectives of this work are listed as follows:

- *Agent definition*: Treat UAVs as autonomous agents. Build corresponding agent models and define relevant parameters. The proposed models are implemented in the Python programming language.
- *Precision farming system architecture proposal*: Build the precision farming system as a multi-agent system. The PFS should have an improved federal architecture, taking advantages of absolute-centralized architecture and standard federal architecture.
- *Multi-objective optimization mapping*: Treat mission planning problem as a multi-objective optimization problem. The relevant optimization objectives should be defined in mathematical equations, considering both the benefit and cost.
- *MP-PSOGA implementation*: Combine the Particle Swarm Optimization and Genetic Algorithms. The main body of MP-PSOGA is based on Particle Swarm Optimization, embedded with crossover and mutation operators from the Genetic Algorithms.
- *Negotiation mechanism design*: After a mission is established, all the agent candidates are competing for taking over the mission through an auction. Each agent candidate places the bid on its most-wanted task, and then, the auction holder will calculate the comprehensive benefit by running MP-PSOGA. After the calculation, the mission will be assigned to the most appropriate agents and these agents will form an agent coalition to carry on the mission cooperatively. Thus, the mission planning strategies for agricultural missions are generated.
- *Simulation validation*: The proposed precision farming system and mission planning approach are validated through simulations. A mission called precise pesticide spraying is established and executed by several Unmanned Aerial Vehicles cooperatively.

The paper is organized as follows: in the next section, the architecture of precision farming system is presented, along with definition of agent (UAV) models. In Section 3, we describe the detailed mission planning approach, including the optimization objectives, proposed MP-PSOGA and negotiation mechanism. In Section 4, the simulation and result is explained. The result is analyzed in order to illustrate that the mission planning strategy, generated by proposed approach, could achieve the optimal performance than other alternative strategies. Lastly, we conclude all the contributions and discuss the future work in Section 5.

## 2. Precision Farming System Architecture

Generally, a precision farming system consists of four main modules; including Remote Sensing (RS), Geographic Information System (GIS), Decision Support System (DSS) and Global Positioning System (GPS). RS is used to collect environmental data for the purpose of monitoring the nutritional status of crops and soil [39]. GIS is used to analyze the collected information, including filtering the useful information from all the data set [40]. DSS is a module that supports organizational decision-making activities for agricultural missions. It is used to make a detailed operation plan for agricultural machineries [41]. GPS is usually employed for guidance of the agricultural machineries [42]. The main concern of our work focuses on the decision support system, which involves the mission planning process.

**Figure 1:**
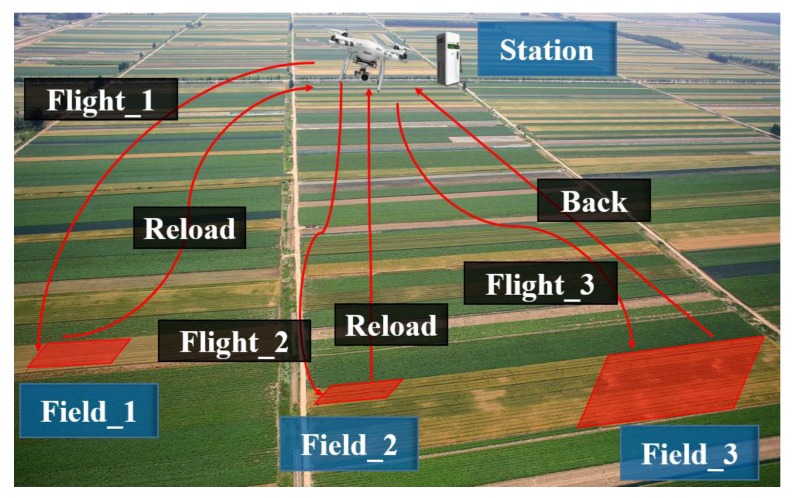
Mission example with single UAV.

The PFS is required to solve complex agricultural missions. Usually, a complex mission is too difficult for a single UAV to complete. For example, a mission called precise pesticide spraying has been established (as shown in Figure 1). The target fields are marked in red and need certain quantities of specific pesticide. It is assumed that only one UAV, equipped with a pesticide sprayer and a pesticide tank, is carrying on such mission. However, the UAV has to fly over the target field to spray the pesticide and fly back to the station for reloading more pesticide, because of the limitation imposed by the maximum capacity. Obviously, a single UAV can hardly complete the mission. Meanwhile, the UAV will consume energy at a fast pace, cause high equipment loss and take high flying risk. Consequently, this is definitely not the ideal plan for the precise pesticide spraying mission. 

However, if there are several UAVs carrying out complex agricultural missions cooperatively, the efficiency could be greatly increased and the cost could be decreased at the same time [43]. All the UAVs in precision farming system are regarded as agents. The agents have characteristics like autonomy, reactivity, pro-activeness and social ability. Autonomy implies that an agent can take actions without external interferences and it has the ability to control its behavior and internal status. Reactivity means that an agent can give certain feedbacks after perceiving the environment. Pro-activity represents that an agent would not only respond to the external environment, but also take direct actions according to its objectives. Social ability describes how an agent can communicate with each other or with human beings. Thus, the agents could construct a multi-agent system and work cooperatively. Complex agricultural missions could be divided into several simple tasks. These tasks will then be assigned to the most appropriate UAVs. Each UAV will fly over to the assigned target fields and spray the pesticide.

Regarding the definition, multi-agent system is a computerized system, consisting of multiple autonomous or semi-autonomous intelligent agents within a certain environment. Through the communication and interaction of these intelligent agents, the multi-agent system [44] has the capability of solving complex problems that a single agent cannot solve, such as mission planning problem in precision farming system. What is more, a multi-agent system has the following characteristics [45].

- *Collaboration*: The agents in multi-agent system are supposed to work with each other cooperatively and solve complex problems that an individual agent cannot handle.
- *Parallelism*: The agents in multi-agent system are able to work in parallel, which increases the efficiency of solving the problems.
- *Robustness*: The multi-agent system does not rely on a single agent and will not be paralyzed because of one failure from an individual agent.
- *Extension*: Extra agents could be added into the multi-agent system and useless agents could be removed from the network. These characteristics increase the reusability and scalability of the system.
- *Distribution*: The data and resources of the multi-agent system are separately stored in each agent. Meanwhile, each agent could be deployed in different physical locations. Agents may share data and resources via specified communication approach, like Agent Communication Language (ACL). The most popular ACLs are Knowledge Query Manipulation Language (KQML) and FIPA Agent Communication Language (FIPA-ACL). Meanwhile, some researchers have made great contributions to develop agent-based simulator for testing communication protocols in precision farming systems [46].

Due to their characteristics and merits, multi-agent systems have been applied to many areas, such as industry, military, finance, agriculture, etc. In our proposal, the multi-agent system is applied to intelligent agriculture.

### 2.1. An Improved Federal Architecture

**Figure 2:**
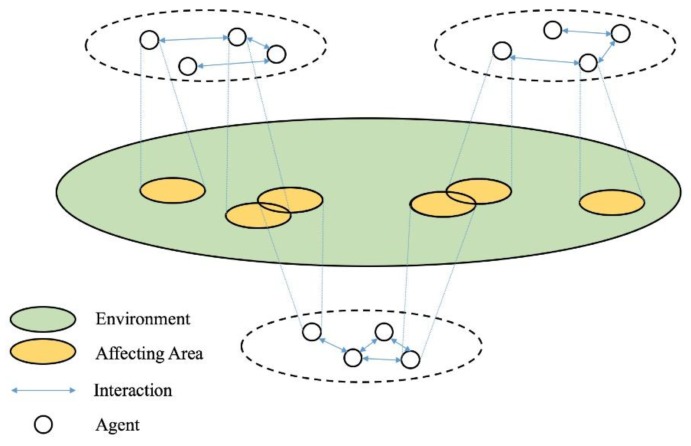
The standard framework of a multi-agent system.

The core of a multi-agent system is to merge all the agents’ capabilities. When several agents act as a complete system, the performance of this system is far beyond that of these agents. The framework of a multi-agent system represents the relationship of the agents. Meanwhile, the framework also indicates the distribution solution, information storage or sharing method. A standard framework for multi-agent systems is shown in Figure 2. In this framework, each agent has its own affecting area that may be overlapped. The overlapped areas reflect the relation of agents’ activities, so it is necessary to establish a certain architecture for the purpose of coordinating these agents [47].

**Figure 3:**
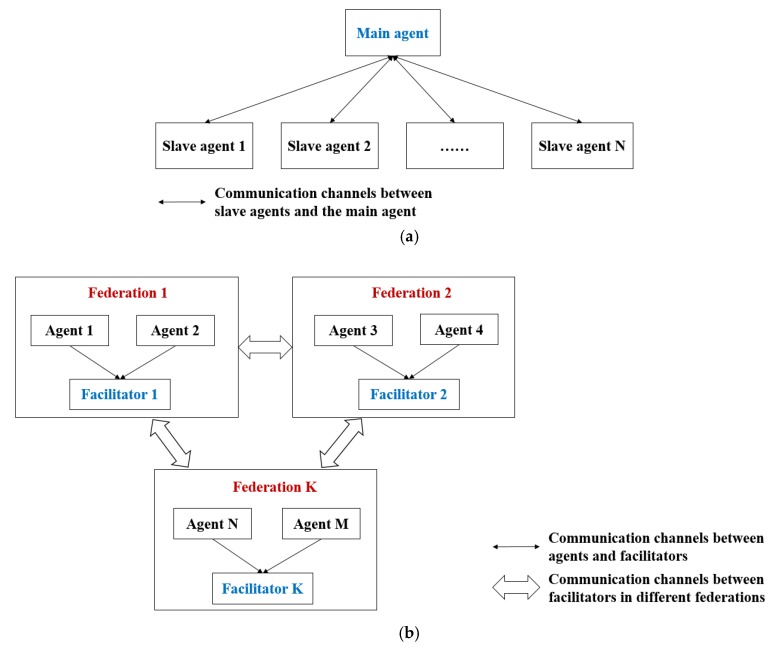
Standard architectures of MAS: (**a**) Absolute-centralized architecture; (**b**) Federal architecture.

Several standard architectures are widely used in the multi-agent system, including the absolute-centralized, absolute-distributed and federal architectures. Additionally, the federal architecture is also known as region architecture. In this paper, we propose an improved federal architecture, taking advantages of absolute-centralized and federal architectures. The mentioned two standard architectures are shown in Figure 3.

In an absolute-centralized architecture, there is only one master agent, with absolute authority, managing the belonging slave agents. The master agent is responsible to data transmission, communication channel, decision-making, etc. The slave agents are supposed to follow the commands and perform required operations. This architecture could decrease the complexity of the system and reduce communication cost. However, its main drawback is that the master agent must have a strong capability, because it is impossible for the master agent to maintain the whole system when there are too many slave agents or the activities of slave agents are too complex. Hence, the absolute-centralized architecture is not applicable to a dynamic and large-scale system, such as precision farming system.

In a federal architecture, the multi-agent system is composed of several federations (regions). Each federation is composed of one facilitator and several normal agents. The facilitators are responsible to communications between each federation. The normal agents are responsible to follow the commands and perform required operations. The merits of such architecture is high flexibility and easy scalability. However, federal architecture lacks of a decision-making terminal.

**Figure 4:**
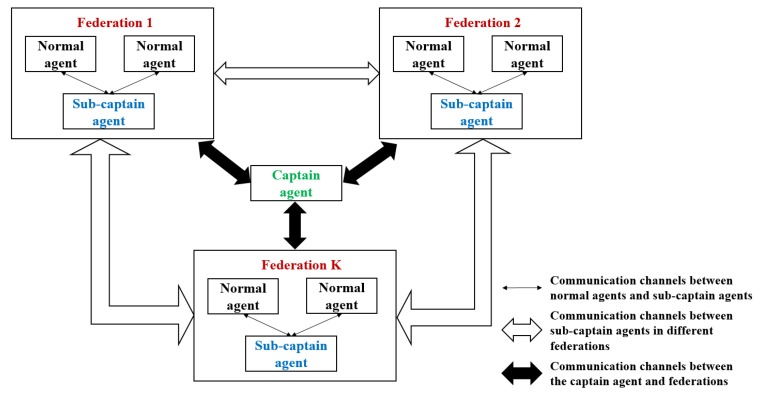
An improved architecture for precision farming system.

Hence, in our proposal, we took the advantages of absolute-centralized and federal architectures and merged these two architectures into an improved federal architecture, shown in Figure 4.

The improved federal architecture has a three-level hierarchy, including one captain-agent, several sub-captain agents and normal agents. The captain agent has the highest priority. Its responsibility is to establish agricultural missions and manage belonging federations. The sub-captain agents, as facilitators in the federations, are responsible to manage communication and transmit assigned tasks to normal agents. The normal agents only have to follow the commands to perform required operations and report real-time status to the sub-captain agent. Additionally, normal agents in different federations cannot communicate with each other directly, the communication has to go through the sub-captain agents. With the improved federal architecture, the precision farming system has the merits of high flexibility, high intelligence, easy scalability and low communication cost. The detail of these three agents will be explained in the next sub-section.

### 2.2. Agents in Precision Farming System

**Figure 5:**
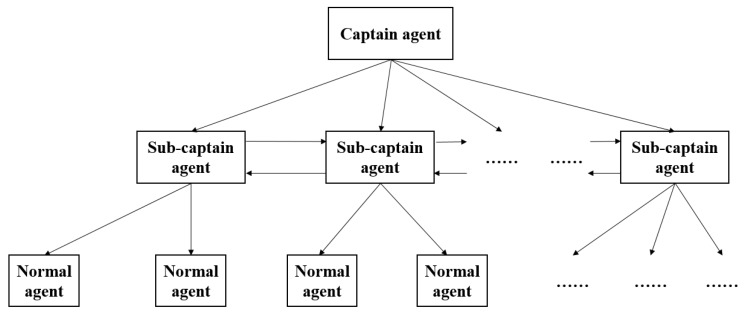
The hierarchy of precision farming system.

As is mentioned in Section 2.1, three kinds of agents are introduced to the precision farming system. Generally, the captain agent is the control center or control terminal, managed by famers or agricultural experts. The sub-captain agent is a movable or fixed communication base. The normal agent is common agricultural machinery, such as a harvester, a UAV, unmanned ground vehicle, and etc. In our case, the normal agents are UAVs. The hierarchy of all three agents is shown in Figure 5. 

### 2.3. Model Definition

To implement the precision farming system, we defined two basic models, including a general target model and a UAV model. The general target model refers to a target farming field, and the UAV model refers to an agricultural machinery responsible for executing assigned tasks. This sub-section explains the detail of these two models.

● General target model

The target in precision farming system may refer to a specific farming area or livestock. For now, we only concerns the static targets, farming areas, in our case. The target model is implemented in Python code and its attributes are stored in an array, called “Target_msg”. Since we are not focusing on planning the flying route of UAV in the target area, the target area is simply presented as a point by coordinate (*x*, *y*). The detail of the array is listed in Table 1.

In the above table, the attribute of “Target_position” represents the location of the target, “Required _resource” represent the demand for certain quantities of resources and “Required_priority” represents the implementation order of the target. The target with higher priority will be handled in the first place. Additionally, the target model has an attribute called “Target_condition”, representing that whether the target is handled completely or not. 

● UAV model

UAV is regarded as normal agents in the precision farming system. The UAV model is implemented in Python code as well and the attributes are stored in an array, called “UAV_msg”. The detail of the array is listed in Table 2.

**Figure 6:**
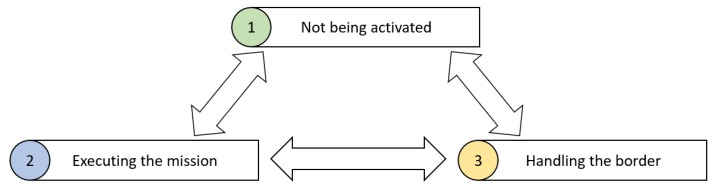
The status of a UAV.

In the above table, the attribute of “UAV_position” represents the current position of UAV, “Phi” represents the current heading angle, “Velocity” represents the flying speed of UAV, “R_min” represents the minimum turning radius of UAV, “Detect_scope” represents the range that a UAV could detect, “Loaded_resource” represents the loading capacity, “Remaining_energy” represents the excess of energy, “Energy_consumption_rate” represents the consumption rate per hour, “Equipment status” represents the status of equipment and “Equipment_loss_rate” represents wear rate per hour [48]. Additionally, it is worthy considering that UAVs may fly out of the range of the farming land. If this happens, the UAVs have to handle the border problem and fly back to the farming land. Hence, the status of a UAV is shown in Figure 6.

In Figure 6, the first status is called “Not being activated”, representing that the UAV does not receive any commands and is not being used right now. The second status is called “Executing the mission”, representing that the UAV is being activated and receives a command. The third status is called “Handling the border”, representing that the UAV may fly out of the range very soon. The UAV has to adjust its flying route by calculating the turning radius. After handling the border problem, the UAV will turn its status back to the previous one. For further implementation, more attributes could be introduced into the general target and the UAV model. For now, we have just proposed the simple models for the purpose of implementing the precision farming system.

## 3. Multi-Objective Optimization Problem in Precision Farming System

A Multi-objective Optimization Problem (MOP), also known as multi-objective programming problem, is concerned with mathematical computations. MOP studies the approaches of optimizing multiple objective functions simultaneously in a given time interval, in order to fulfill the requirement of several criteria [49,50]. It is important to mention that these multiple objectives of MOP have certain constraints and are always conflict with each other.

The multi-agent system has been studied by lots of researchers, so various approaches have been developed in order to solve the MOP. Generally, there are two types of approaches. The first one is based on linear objectives optimization. This approach turns the multiple objectives into a single objective by the linear weighted sum equation. However, this approach could not achieve a precise result when the system is dynamic and in large scale. Furthermore, this approach only figures out a single result, which is a valid solution among the non-inferior set. The second approach is to compute the solutions in parallel based on the computation intelligence. In computation intelligence, the most popular algorithms are Genetic Algorithms (GAs) and Particle Swarm Optimization (PSO). These two algorithms are studied in this paper and combined into an improved algorithm, MP-PSOGA.

### 3.1. Problem Statement

The mission planning problem in SWARMs project [31] is treated as a multi-objective optimization problem and it considers two optimization objectives, including completion time of the mission and energy consumption of robots. However, it is not thoughtful enough to only consider these two objectives. Extra optimization objectives should be added in order to generate the optimal mission plan.

Mission planning in precision farming system is a multi-objective optimization problem too. The completion of the agricultural mission is evaluated by several criteria, including the completion time, achieved benefit, energy consumption and equipment loss. However, these criteria are expected to be always contradicting in their objectives. For example, if a farmer wants to complete the agricultural mission in a short time, though the completion time is decreased, the energy consumption and equipment loss of agricultural machineries may be increased. Or the farmer wants to complete the mission with low energy consumption, the completion time has to be increased. Hence, the evaluation criteria in precision farming system are treated as optimization objectives. 

### 3.2. Optimization Objectives

In the precision farming system, the completion time and expected benefit are treated as the beneficial objective. The equipment loss and energy consumption are treated as the cost objective. In order to define these two objective functions, a target allocation matrix is given in advance. Assuming that the number of UAVs is $N=\left\{V_{1},V_{2},V_{3},\ldots ,V_{N}\right\}$, and the number of targets is $M=\left\{T_{1},T_{2},T_{3},\ldots ,T_{M}\right\}$. The target allocation matrix, $X^{N*M}$, is defined as follows:

(1) $$x_{ij}=\{\begin{array}{c} 1,T_{j}\;is\;assigned\;to\;V_{i}\\ 0,\;T_{j}\;is\;assigned\;to\;V_{i} \end{array}$$

In the above equation, i=1,2,3,…,N and j=1,2,3,…,M. The target allocation matrix represents whether a UAV is assigned with a target or not.

During the mission planning process, multiple targets may be assigned to a single UAV, and a single target may be assigned to multiple UAVs as well. For the purpose of balancing the payload, two constraints should be established as follows:

(2) $$\{\begin{array}{c} \sum_{i=1}^{N}x_{ij}\leq N_{max}\\ \sum_{j=1}^{M}x_{ij}\leq M_{max} \end{array}$$

In the above equation, the parameter of $N_{max}$ represents the maximum number of the UAVs in a single target, and $M_{max}$ represents the maximum number of the target that is assigned to a single UAV. Meanwhile, for the stability of the precision farming system, the threshold should be established as follows:

(3) $$\{\begin{array}{c} \sum_{i=1}^{N}\left|\frac{1}{N}\sum_{i=1}^{N}\sum_{j=1}^{M}X_{ij}-\sum_{j=1}^{M}X_{ij}\right|\leq \alpha \\ \sum_{j=1}^{M}\left|\frac{1}{M}\sum_{i=1}^{N}\sum_{j=1}^{M}X_{ij}-\sum_{i=1}^{N}X_{ij}\right|\leq \beta \end{array}$$

In the above equation, the parameters of α and β represent the thresholds of the precision farming system. The thresholds could be set by the agricultural expert or the user.

#### 3.2.1. Beneficial Objective

Before deciding to accept or refuse the assigned mission, the UAVs have to calculate the economic benefit. If the calculation result is greater than the expected benefit, the UAV shall consider to join the corresponding coalition to carry on the mission. Otherwise, the UAV may refuse to join the coalition. Hence, the beneficial objective function is defined like this:

(4) $$B\left(X\right)=\sum_{j=1}^{M}\pi_{j}*P_{j}$$

In the above equation, the parameter of $\pi_{j}$ is the probability function of handling with the target, $T_{j}$, and $P_{j}$ is the target priority. The probability function of handling the target, $\pi_{j}$, is defined as:

(5) $$\pi_{j}=1-\prod_{i=1}^{M}\left(1-x_{ij}*\;Pob_{ij}\right)$$

In the above equation, the parameter of $Pob_{ij}$ is the efficiency function, representing the efficiency of UAV to handle the target.

In the beneficial objective function, the target priority, $P_{j}$, is considered. This parameter represents the implementation order by the UAVs. The target with a higher priority will be implemented first. 

#### 3.2.2. Cost Objective

When the UAVs are performing required operations, they have to consume a certain amount of energy, such as fuel or electricity from a battery. Meanwhile, the UAV has certain mechanical loss due to the equipment wear. Furthermore, the value of UAV itself should be taken into consideration, because it is not absolutely safe during the mission execution. There is a probability that the agricultural equipment becomes damaged (for example, if a UAV is hit accidently by a bird and then crashed). 

The value of UAV is decided by the value of its equipment, like sensors, cameras, actuators and etc. Hence, the cost objective function is defined as follows:

(6) $$C\left(X\right)=\sum_{i=1}^{M}\gamma_{i}+\rho_{i}*V_{i}$$

In the above equation, $\gamma_{i}$ is the cost probability function of UAV, $\rho_{i}$ is the damage probability function and $V_{i}$ is the value of UAV itself. The cost probability function is defined as follows:

(7) $$\gamma_{i}=\;\sum_{j=1}^{M}L_{i}\left(T_{j}\right)*x_{ij}$$

In the above equation, $L_{i}\left(T_{j}\right)$ is the cost function. It considers the energy consumption and equipment loss during the mission execution. The retail of this function is described as follows:

(8) $$L_{i}\left(T_{j}\right)=f\left(Cost_{ij}^{engCons},Cost_{ij}^{equLoss}\right)$$

In the cost function, the parameter of $Cost_{ij}^{engCons}$ represents the energy consumption of the UAV and $Cost_{ij}^{equLoss}$ represents the equipment loss. In addition, the cost function could be modified according to extra criteria.

### 3.3. Proposed MP-PSOGA

As mentioned at the beginning of Section 3, Particle Swarm Optimization and Genetic Algorithms are used to solve the multi-objective optimization problem. However, these two common algorithms have their own advantages and disadvantages. In precision farming system, the optimization objectives have been defined. The next step is to develop an improved algorithm to solve the mission planning problem. In this paper, we combined the Particle Swarm Optimization and Genetic Algorithms into MP-PSOGA.

#### 3.3.1. Feasibility of Combining Particle Swarm Optimization and Genetic Algorithms

**Figure 7:**
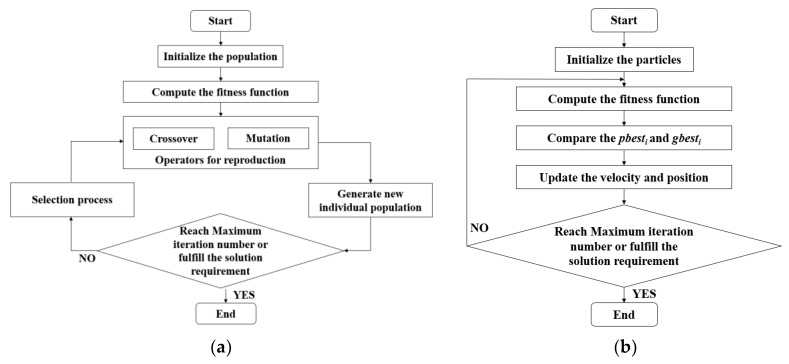
Flow chart: (**a**) Genetic Algorithms; (**b**) Particle Swarm Optimization.

The Genetic Algorithms are based on biological natural selection and random searching of genetic mechanism. Differing from the traditional searching algorithms, the Genetic Algorithms start the searching process from a randomly-generated initial set, called population. In the population, each individual is one solution, called chromosome. A chromosome is a chain of characters, such as binary string. The process, which describes the evolutionary of the chromosomes, is called heredity. During each iteration. The fitness function is calculated to evaluate the performance of the current chromosomes. For the next iteration, a new generation of the chromosomes is born, called offspring. The new chromosomes are generated by the process of crossover and mutation from the previous chromosomes. In the new population, the chromosomes are selected according to the fitness function, which means that some bad chromosomes will be eliminated. The chromosomes with higher fitness value have a greater chance of the selection. The number of the population remains at a constant number after the process of the crossover or the mutation. Lastly, the population will converge on the optimal chromosomes after several iterations. This population is called the Pareto optimal or the Pareto sub-optimal. The flow chart of Genetic Algorithms are shown in Figure 7a. The Genetic Algorithms are used in the SWARMs project and it is able to generate mission plans successfully.

The Particle Swarm Optimization is inspired by the behavior of foraging. The swarm consists of plenty of particles. The PSO relies on the communication of these particles in order to revolutionize together. These particles have the capability of recording their own best known positions, and comparing the local best positions with the global best position. After finding a better position, a particle would adjust its route and gather towards to the global best position. In multi-objective optimization problem, a global best position represents the optimal value of the fitness function, and this optimal value is the Pareto optimal solution. The flow chart of Particle Swarm Optimization is shown in Figure 7b.

Before combining the Particle Swarm Optimization and Genetic Algorithms, it is essential to analyze these two algorithms [51,52,53,54]. These two algorithms have their own advantages and disadvantages. The advantages of Genetic Algorithms could be summarized as the following:

1) The Genetic Algorithms do not require a precise mathematical model, because GAs do not need to know the detail of the optimization problem. The characteristics of evolution allow GAs to deal with the objective functions and constraints in any format.
2) The traditional optimization method always compares two neighbor values in order to achieve the solution convergence. However, the genetic operators of GAs search in all the dimensions for the global optimal solution.
3) Within the Genetic Algorithms, several generations could be constructed at the same time, which means that the computation process could be distributed and ran in parallel. Furthermore, since several generations could be constructed, it is possible to extend GAs and combine it with other optimization algorithms for the purpose of improving the performance.

The disadvantages of Genetic Algorithms are:

1) The encoding process of Genetic Algorithms are complex. Meanwhile, the result has to be decoded for readability after the computation.
2) The crossover and mutation probabilities have great influence on the performance of Genetic Algorithms. Generally, these probabilities are set according to the experts’ experience. However, this could lead to longer computation time for finding the Pareto optimal in specific application background.
3) The performance of Genetic Algorithms relies on the initial population. If the population is generated in bad quality, it may lead to longer time for crossover and mutation process to generate qualified chromosomes.

The advantages of Particle Swarm Optimization could be summarized as:

1) The performance of Particle Swarm Optimization does not rely on the initial population. No matter how many particles in the initial swarm, the performance of PSO remains steady.
2) The particles have a memory storage where stores historical local best position. Furthermore, the particles could learn from the experience and adjust the searching strategy for a quicker convergence to the Pareto optimal.
3) The encoding process for Particle Swarm Optimization is much easier than that of Genetic Algorithms. The particles in the swarm only need to control their velocity and flying direction during the searching process. This method is based on the velocity-movement model.

The disadvantages of Particle Swarm Optimization could be summarized as follows:

1) The search process may be easily trapped at a local best position and stop searching the global best position. This is due to inappropriate settings of parameters in the velocity updating equation.
2) The convergence speed is slow in the later stage and quality of the convergence lacks of precision.
3) The Particle Swarm Optimization requires a precise mathematical model, which means that PSO needs to understand the detail of the optimization problem. Each parameter should be exactly corresponding to the objectives of the problem.

After analyzing the advantages and disadvantages of PSO and GAs, the common grounds and differences of both algorithms are concluded. In both algorithms, the initial population is generated randomly. Then, the candidate solutions are evaluated by the fitness function. Meanwhile, the termination condition is almost the same. Regarding the differences of these two algorithms, comparing with GAs, PSO has a different method of sharing information. In PSO, all the particles are gathering towards to the global best position. The local best positions are only stored by the particle itself and will not be shared with other particles. However, in GAs, the chromosomes are willing to share information with each other, not only the useful fragments, but also the useless fragments.

In this paper, we proposed an improved optimization algorithm, MP-PSOGA, by combining PSO and GAs. The MP-PSOGA takes advantages of both algorithms and requires a shorter computation time for convergence. The detail of MP-PSOGA is explained in the following section.

#### 3.3.2. Dynamic Parameters in MP-PSOGA

In order to combine Particle Swarm Optimization and Genetic Algorithms, several parameters should be defined in advance, including weight and learning factors in velocity updating equation, crossover and mutation probabilities in genetic operators. Differing from the previous work [55], some researchers treat the parameters of decision-making process as fixed values or pre-set intervals, in our work, we treat the parameters with dynamic updating mechanism. With the help of such dynamic updating mechanism, the MP-PSOGA could achieve better performance.

Firstly, the parameter of weight in velocity updating equation has great influence on the searching capability of the particles and the convergence speed. A higher weight has the advantage of increasing the capability of searching the global best position. However, it may decrease the capability of searching the local best position. A lower weight has the merit of finding a precise optimal position, but it may cost longer computation time and decrease the convergence speed. Hence, it is necessary to use a dynamic weight in order to achieve a balance between the searching capability and the convergence speed. The dynamic weight updating equation is defined as follows:

(9) $$W=\{\begin{array}{c} W_{min}-\frac{\left(W_{max}-W_{min}\right)*\left(f-f_{min}\right)}{f_{avg}-f_{min}}\\ W_{max},f>f_{avg} \end{array},f\leq f_{avg}$$

In Equation (9), $W_{max}$ is the maximum value of the weight and $W_{min}$ is the minimum value of the weight. Normally, $W_{max}$ is set at 0.9 and $W_{min}$ is set at 0.4. The parameter of f represents the current fitness value. The parameters of $f_{avg}$ and $f_{min}$ represent the current average fitness value and the minimum fitness value respectively.

Secondly, the learning factors, $C_{1}$ and $C_{2}$, have influence on the movement trajectory of the particles. If $C_{1}$ and $C_{2}$ choose the inappropriate values, the particles may wander in a local area and converge to a small value. Therefore, choosing appropriate values for the learning factors helps PSO to achieve a better convergence precision and increase the searching capability. The proposed dynamic learning factor updating equation is defined as follows:

(10) $$\{\begin{array}{c} C_{1}=C_{1,s}+\frac{C_{1,e}-C_{1,s}}{t_{max}}*t\\ C_{2}=C_{2,s}+\frac{C_{2,e}-C_{2,s}}{t_{max}}*t \end{array}$$

In Equation (10), the parameters of $C_{1,\;s}$ and $C_{2,s}$ represent the initial value of the learning factors. The parameters of $C_{1,e}$ and $C_{2,e}$ represent the value in current iteration. The parameter of t represents the current iteration round and $t_{max}$ represents the maximum iteration round. Normally, $C_{1,s}$ and $C_{2,s}$ are set at 2.5, and $C_{1,e}$ and $C_{2,e}$ are set at 0.5.

Thirdly, the crossover and mutation are the key processes of generating new child solutions in Genetic Algorithms. These two operators are introduced into Particle Swarm Optimization in order to avoid early convergence and help the particles to jump out of the local best positions. However, fixed crossover and mutation probabilities may not ensure finding the global optimal solution. Hence, dynamic crossover and mutation probability updating equations are defined as follows:

(11) $$P_{c}=\{\begin{array}{c} P_{c1}-\frac{\left(P_{c1}-P_{c2}\right)*\left(f^{\prime }-f_{avg}\right)}{f_{max}-f_{avg}},f^{\prime }\geq f_{avg}\\ P_{c1}\;,f^{\prime }<f_{avg} \end{array}$$

(12) $$P_{m}=\{\begin{array}{c} P_{m1}-\frac{\left(P_{c1}-P_{c2}\right)*\left(f^{\prime }-f_{avg}\right)}{f_{max}-f_{avg}},f\geq f_{avg}\\ P_{m1}\;,f<f_{avg} \end{array}$$

In Equations (11) and (12), the parameters of $P_{c1}$ and $P_{m1}$ represent the maximum values of crossover and mutation probabilities. The parameters of $f_{max}$ and $f_{avg}$ represent the maximum fitness value and average fitness value respectively. The parameters of $f^{\prime }$ and f represent the fitness value of local best solution and current solution respectively. Normally, $P_{c1}$ is set at 0.9, $P_{c2}$ is set at 0.6, $P_{m1}$ is set at 0.1 and $P_{m2}$ is set at 0.001.

#### 3.3.3. Principle of MP-PSOGA

**Figure 8:**
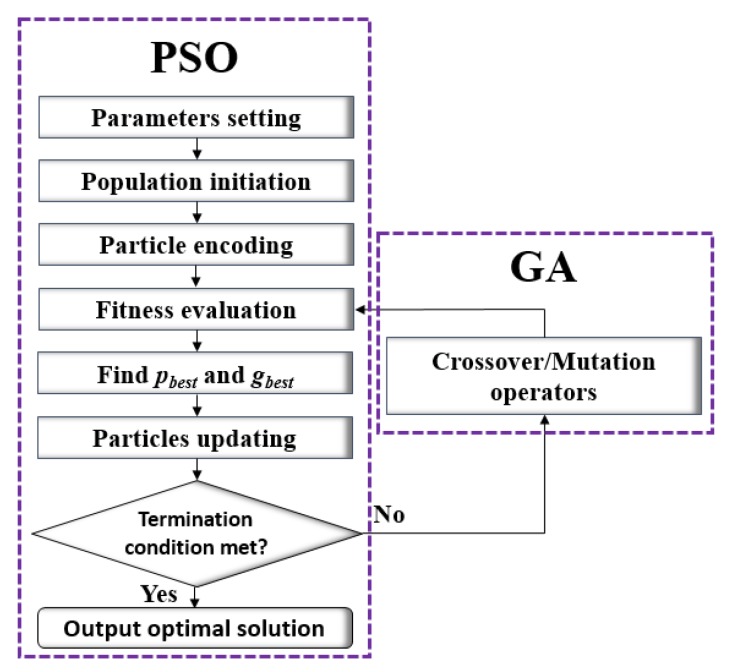
Flow chart of MP-PSOGA.

The main body of MP-PSOGA is based on Particle Swarm Optimization, embedded with crossover and mutation operators from Genetic Algorithms. The flow chart of MP-PSOGA is shown in Figure 8. As shown, MP-PSOGA firstly initializes the parameters, including weight, learning factors, crossover and mutation probabilities. Secondly, the particles are initialized and encoded with a starting position, velocity and heading direction. Thirdly, the fitness value of all the particles are calculated for evaluation. The global best position and local best positions are chosen based on the evaluation. Fourthly, each particle updates their position and parameters of MP-PSOGA is updated as well according to Equations (9)–(12). Fifthly, several particles with bad fitness values are selected for crossover and mutation processes. Newly-generated particles are re-evaluated. The particles keep updating their positions as the iteration keeps going, until the termination condition is met. The termination condition usually is to reach the maximum iteration number or the Pareto optimal is found.

**Figure 9:**
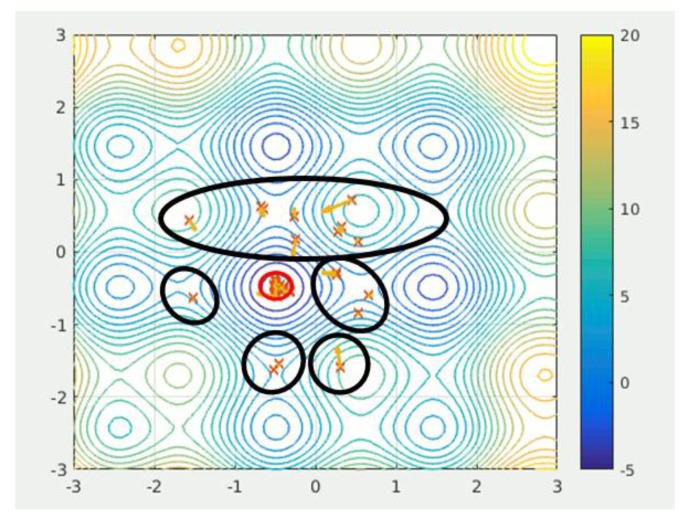
Bad fitness value particles in the swarm. Adopted from Wikipedia [56].

During the iteration, some particles, with bad fitness value, cannot find the global optimal. These particles are marked in black circle in Figure 9 [56].

In Figure 9, these bad particles may take a long time to find the global optimal or even cannot find the global optimal. In order to improve these particles, the crossover and mutation operators from Genetic Algorithms are introduced. 

**Figure 10:**
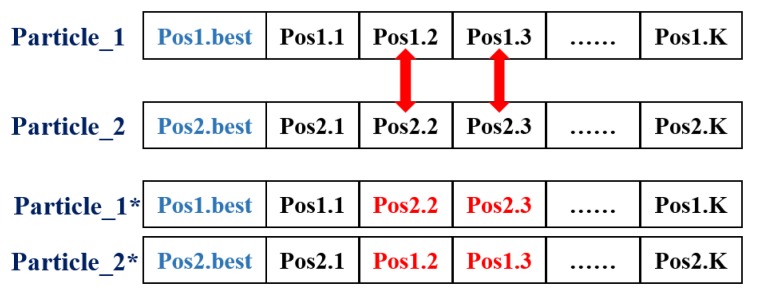
An example of crossover process.

Regarding the crossover operator, several pairs of bad particles are selected to exchange their historical flying data. In order to explain the detail of crossover operator, an example is shown in Figure 10.

In Figure 10, “Particle_1” and “Particle_2” are selected, assuming that these two particles have bad fitness value. The crossover operator indicates that these two particles should exchange the second and third historical flying data. After the crossover process, two new particles, “Particle_1*” and “Particle_2*”, are generated. Since these two particles already know that the exchanged positions are not the global optimal ones, they will not waste time to flying to such positions. Instead, they will search for other unknown positions instead. The crossover operator enables to increase the convergence speed of MP-PSOGA.

Regarding the mutation operator, several particles, with bad fitness value, are selected and removed from the swarm. In MP-PSOGA, the particles with last five worst fitness value are selected and removed. However, the number of particles should remain unchanged in order to keep the stability of the swarm. So, five newly-generated particles are added into the swarm, replacing the removed bad particles. Additionally, the new particles are randomly generated with initialized position, velocity and heading directions. The detail of mutation operator may refer to Section 4 of Branko et al. paper [31]. Four kinds of mutations are introduced, including the swap mutation, replace mutation, shrink mutation and growth mutation. The crossover and mutation process is performed once in each iteration. These two operators enable the MP-PSOGA to increase the evolution speed and avoid precocity.

### 3.4. Mission Planning Approach

The purpose of the proposed mission planning approach is to distribute the established mission to the most appropriate agents. By this approach, the precision farming system could achieve the maximum economic benefit with reasonable resources. The mission planning strategy is generated by MP-PSOGA. However, before a mission is distributed, several operations should be processed before assigning this mission to UAVs, including mission decomposition, task priority sorting, negotiation process and etc. 

#### 3.4.1. Mission Decomposition

An agricultural mission is usually too complex for the UAVs. At this time, this mission has to be decompiled. The decomposition process aims to decomposing one mission to several minor tasks, which could be operated in parallel. These tasks are not conflict with each other. There are two general methods of decomposing the mission, centralized and distributed decomposition [57,58]. 

The centralized decomposition is usually implemented by pre-allocation or Trader allocation. The approach of pre-allocation decomposes the mission according to fixed principles defined by the users. The approach of Trader allocation decomposes the mission according to the capability list of the agents. However, these two approaches are lack of flexibility and cannot adapt to the dynamic environment. Hence, the centralized decomposition is not applicable to the precision farming system.

The distributed decomposition is usually implemented by contract net protocol (CNP) or acquaintance coalition network (ACN). CNP decomposes the tasks through communication within the multi-agent system. ACN decomposes the mission by searching capability matrix. 

**Figure 11:**
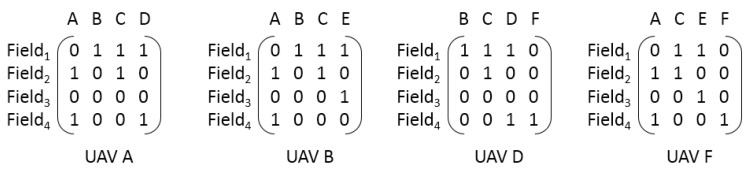
Part of the capability matrix of agent candidate.

In precision farming system, it is assumed that a mission called pesticide spraying is established. The mission requires six unmanned aerial vehicles to spray pesticide over four specific farming fields. Each UAV is able to load certain quantities of pesticide and each farming field requires certain quantities of pesticide. Hence, the mission is decomposed into four tasks, “Spraying pesticide over field 1”, “Spraying pesticide over field 2” and so on. According to CAN, part of the capability matrix is given in Figure 11.

**Figure 12:**
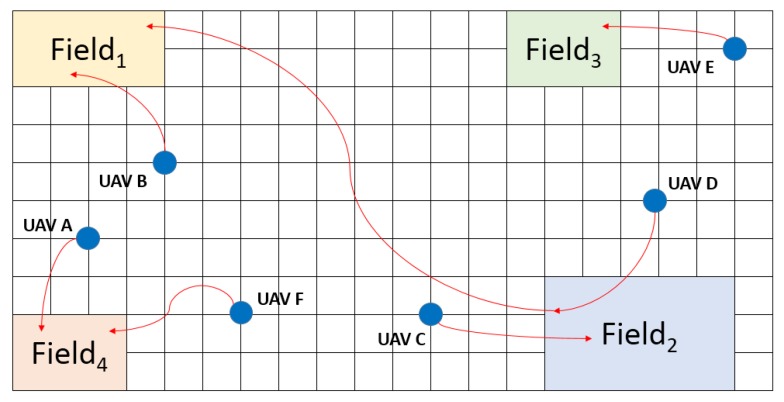
The result of mission decomposition and one possible mission planning strategy.

As is shown in Figure 11, the acquaintance of UAV A is UAV B, C and D, the acquaintance of UAV B is UAV A, C and E, the acquaintance of UAV D is UAV B, C and F, the acquaintance of UAV F is UAV A, C and E. Meanwhile, the value of one represents that the corresponding UAV is able to carry on the pesticide spraying task in field 1, the value of zero represents that the corresponding UAV is unable to carry on such task. As is mentioned before, one task could be took over by several agents, and one agent could take over several tasks. So, the result of mission decomposition and one possible mission planning strategy is shown in Figure 12.

The above figure illustrates that field 1 is took over by UAV B and D, field 2 is took over by UAV C and D, field 3 is took over by UAV E, and field 4 is took over by UAV A and F. The mission decomposition process is able to decrease the complexity of computing the Pareto optimal in the later stage, because the MP-PSOGA only needs to compute the Pareto optimal for each task.

#### 3.4.2. Task Priority Sorting

In most task priority sorting cases, several approaches are used, such as earliest deadline first (EDF) and least slack first (LSF) [59,60]. Generally, the task priority is decided by several parameters, including deadline, spare time, task importance and etc.

However, to keep it simple in the precision farming system, the task priority is sorted by the number of the token that a task has. The task is defined through the general target model. In Section 2.3, the general target model has the attribute, “Required_priority”. The target with more tokens has the higher priority and will be handled first. Additionally, if one UAV chooses to carry on two or more tasks, the UAV will handle the task with more tokens first. 

#### 3.4.3. Negotiation Process

The communications in multi-agent system enables the agents to negotiate with each other. The capability of negotiation is one of the key characteristics of the intelligent agents. Nowadays, several approaches are used in the negotiation process, such as auction mechanism, cooperative game theory, non-cooperative game theory and etc. In our work, we used the auction mechanism in the negotiation process.

The auction mechanism is widely used in multi-agent system for task assignment and resource allocation. In precision farming system, the general procedure of the auction mechanism is stated following several steps: the captain agent, as the auctioneer, announces that the task auction begins and generates the auction order for the task according the task priority. The auction order of agents is generated randomly. In the first round of auction, each qualified agent takes turns to place the bid on the task with highest priority. In the second round, qualified agents take turns to place the bid on the next task. After all the rounds are finished, the auctioneer will calculate the comprehensive benefit and generate the most appropriate agent coalitions for tasks. If the termination condition is not met, the auctioneer will hold the second auction and generate a new mission planning strategy. The newly-generated strategy is compared with the strategy, which is generated in the last auction. If the newly-generated strategy dominates the previous one, it will replace the previous strategy and be applied to the precision farming system.

During the negotiation process, the bids from agents should follow the beneficial and cost objective function, which are defined in Section 3.2. The pseudo code of the negotiation process is shown below:

**Algorithm**: Negotiation process   Begin   target_List = targets_Msg(coordinates, needed_resource, priority, …);   agent_List = agent_Msg(coordinates, phi, v, …);   while (the Pareto optimal is not found or the deadline is not reached):      round = 0;      For i in range (0, N):        For j in range (0, M):          Agent[i] computes B(X)[j] and C(X)[j];          Agent[i] generates mwt_List[i], the most-wanted target list;        End for;      End for;      bidding_List = random_List();      For i in bidding_List:        Agent[i] places the bid;        Agent[i] broadcasts its selection;        if (k > i):          Agent[k] updates [B(X), C(X)] by new = (1 − Pij) * [B(X), C(X)];        End if;      End for;      For j in range target_List:;        auction_Host calculates [B(X), C(X)];      End for;      auctioneer accepts the bids and generates the solution;   End while;   End.

In each iteration, a feasible strategy is generated. The current-round strategy is compared with the one from last-round. If the current strategy dominates the previous one, the precision farming system will adapt the current strategy, otherwise, the strategy will remain as the previous one. In this paper, the negotiation result is mainly decided by the auctioneer, not the agent candidates, because it is difficult to distribute the calculations into multiple agents, considering the communication cost.

Additionally, if an agent has remaining resources after placing the bid on the first target, it could place a new bid on the second target as well. 

The final mission planning strategy represents the Pareto optimal one and will be adapted by the precision farming system. The UAVs in PFS will then follow the generated strategy to form the federations. Along with the control terminal (control unit), each component of the multi-agent system is constructed.

#### 3.4.4. Task Execution

**Figure 13:**
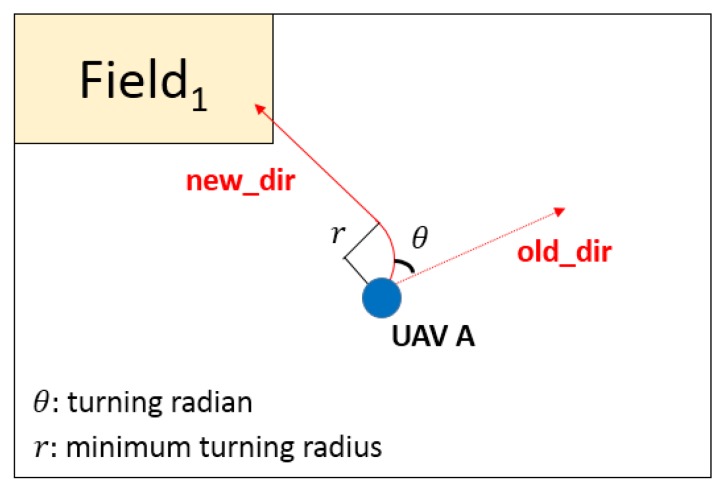
The process of adjusting the heading direction.

After the processes of mission decomposition, task priority sorting and negotiation, the established mission is decomposed to several tasks. These tasks are assigned to the most appropriate agents according to the negotiation result. As one task is assigned to multiple agents, an agent coalition for the task could be formed, aiming to enabling the agents to carry on the task cooperatively. As soon as an agent receives the command to carry on the task, its status shall turn to type 2 “Executing the mission”, according to Section 2.3. Meanwhile, the agent should plan the flying path to the target. Figure 13 displays how the agent is adjusting its heading directions.

In Figure 13, the agent calculates the minimum turning radius based on the current heading direction and target position. The time for adjusting the heading direction is also considered, since the arrival time to the target is one of the evaluation criteria. During the process of executing the task, if the agent is about to fly beyond the border, it should turn its status to type 3 “Handling the border”. Until the completion of handling the border, its status will turn back to type 2 “Executing the mission” and continuing to carry on the task.

#### 3.4.5. Mission Re-Planning

The precision farming system is considered as a dynamic system because of three main factors: new missions are established, UAVs lose the capability of carrying on the current mission, and the meteorological environment is changed.

● New missions are established.

The users of precision farming system have the permissions to establish new missions. For example, the UAVs are carrying on a pesticide spraying mission. In this mission, there are four fields needed to be sprayed. During the run time, the user adds two extra fields into the pesticide spraying mission, so there are six fields needed to be sprayed now. Another option is that the user considers that one of the fields does not need to be sprayed, so this field is removed from the task list; hence there are only three fields to be sprayed now. Any one of these situations will change the mission planning strategy and lead to a mission re-planning process.

● UAVs lose the capability of continuing the on-going mission.

Here, it is assumed that several UAVs are carrying on a monitoring mission in the farming land. Suddenly, one of the machineries has a malfunction problem and is unable to carry on the mission anymore. Under such circumstance, the monitoring mission is impossible to be performed according to the original mission planning strategy, because losing one of the pieces of equipment may lead to an incapacitating lack of resources. Hence, the mission planning strategy has to be re-generated.

● The meteorological environment is changed.

When the UAVs are carrying out a mission, the meteorological environment may change. The meteorological environment has a great influence on the efficiency of machinery. Under a bad weather situation, the completion time of the established mission may be delayed. Meanwhile, in order to avoid the risk of working in bad meteorological environment, UAVs have to accelerate the pace in order to complete the mission in a shorter time. Hence, several requirement of the mission is changed, leading to a mission re-planning process.

**Figure 14:**
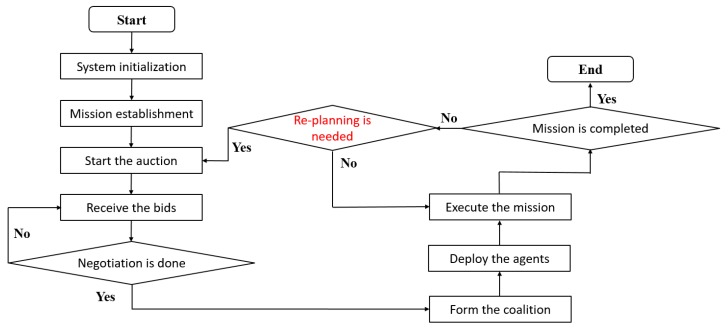
The mission re-planning mechanism.

In Figure 14, the flow chart of the precision farming system with the mission re-planning process is shown. The precision farming system checks the dynamic changes during the run time. If the situation is changed and a mission re-planning process is required, the system will start a new auction and generate an appropriate mission planning strategy to replace the old one. Once the system starts the auction, the rest of procedures remains unchanged: agent candidates places the bids, the auctioneer calculates the comprehensive benefit and generate the mission planning strategy, selected agents execute the command to complete the mission and so on. 

## 4. Simulation and Results

In the simulation, a mission called “pesticide spraying” is established and carried on by six unmanned aerial vehicles cooperatively. Each UAV is equipped with a pesticide tank and a spray nozzle. The mission planning strategy is generated by running MP-PSOGA. The simulation result is analyzed by comparing the Pareto optimal and alternative strategies. 

### 4.1. Simulation Statement

The simulation is designed to carry on a pesticide spraying mission in the precision farming system. The total coverage of the faming field is 2000 × 2000 m^2^, representing by the coordinate (−1000, 1000) in x axis and (−1000, 1000) in y axis respectively. Four types of pesticide are considered in this simulation: *P_1_, P_2_,* and *P_3_*. Three fields in the farming land are needed to be sprayed. Each field requires certain quantities of different pesticide. The three fields are marked as target and have their own positions, represented by coordinates (x, y). The detail of these three fields and required resources are listed in Table 3.

As it is shown in Table 3, three fields are specified and their positions are given. Pesticide types and quantities for each field are specified as well. For example, the first field requires 3 units of pesticide P*_1_*, 5 units of pesticide P*_2_* and 4 units of pesticide P_3_. The spraying task for field 1 should be complete within an hour. The position of the first field is (−350, −200). The number of tokens for the first field is 3. So does the rest of the field.

In the simulation, six unmanned aerial vehicles are supposed to complete such pesticide spraying mission cooperatively. Each UAV has an initial position, velocity, heading directions and so on. Meanwhile, each UAV is able to load certain quantities of different pesticide. The loading detail of these six UAVs is listed in Table 4.

**Figure 15:**
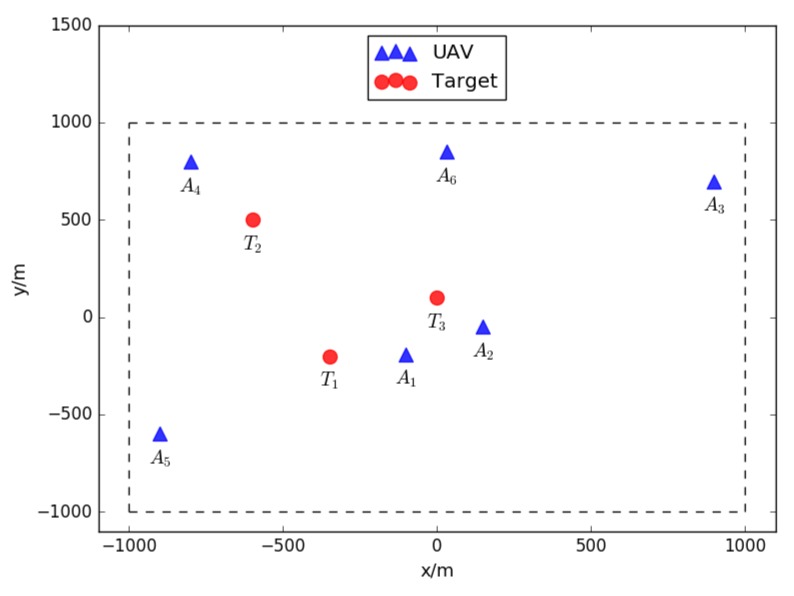
The initial positions of targets and UAVs.

As depicted in Table 4, the first UAV loads two units of pesticide P_1_, two units of pesticide P_2_ and three units of pesticide P_3_. The second UAV loads two units of pesticide P_1_ and one unit of pesticide P_3_. So do the rest of the UAVs. The initial positions of these six UAVs are indicated in Table 4 as well. The initial positions of targets and UAVs are shown in Figure 15. The targets are marked in blue triangles and UAVs are marked in red circles.

Obviously, an individual UAV is unable to spray the required quantity of pesticide over all three fields. Hence, a mission planning strategy is generated for each field by running MP-PSOGA. The selected agents will form the agent coalition in order to complete the task cooperatively.

### 4.2. Parameter Setting

After presenting the information of targets and UAVs, here comes the parameter setting for MP-PSOGA. As is stated in Section 3.2.2, the involving parameters include weight (*W*), learning factors (*C*_1_ and *C*_2_), crossover probability (*P_c_*) and mutation probability (*P_m_*). The initial setting for the parameters is listed in Table 5.

Apart from above mentioned parameters, the size of population is 40 and maximum iteration number is 50. The iteration may stop when the Pareto optimal is found. Otherwise, the output should be the generated strategy in the last iteration. 

The simulation is performed on a Lenovo ThinkPad S2 laptop equipped with an Intel(R) Core(TM) i5-6200U CPU @ 2.30GHz and 8 GB RAM, running the 64-bit Windows 10 operating system. The Python programming language version used is “Python 3.6.3”.

### 4.3. Result

**Figure 16:**
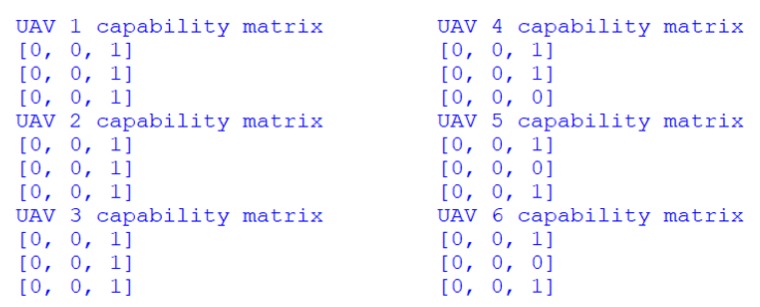
Initialized capability matrixes of all six UAVs.

As it is mentioned in Section 3.4.1, each UAV has its own capability matrix. In this simulation, the acquaintances of each UAV are the two closest-numbered UAVs. For example, the acquaintances of UAV 1 are UAV 2 and 3, the acquaintances of UAV 2 are UAV 1 and 3, and the acquaintances of UAV 3 are UAV 2 and 4. It happens the same with all the other UAVs. Based on needed resources of each target fields, the initialized capability matrixes of all the UAVs are shown in Figure 16.

**Figure 17:**
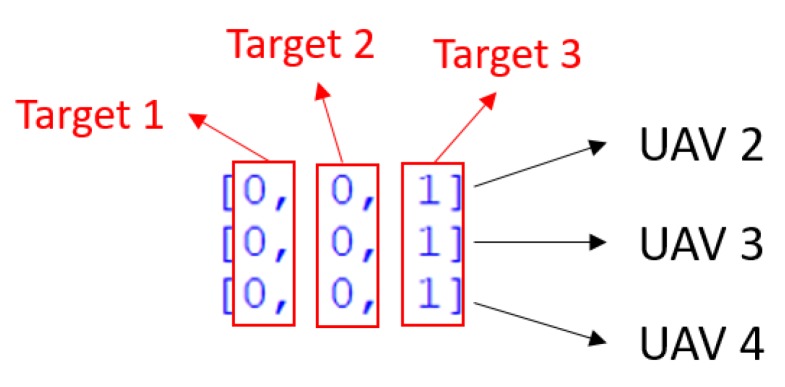
Explanation of capability matrix.

In Figure 16, value 0 represents that the corresponding UAV is not taking over the target and value 1 represents that the corresponding UAV is taking over the target. Each row of the matrix indicates the capability of one UAV, along with the capability of its acquaintances. Each column of the matrix indicates the task candidates. In Figure 17, this is the capability matrix of UAV 3, along with its acquaintances, UAV 2 and 4. All three of these UAVs are capable of taking over target 3.

The format of such capability matrix is explained as follows:

As mentioned in Section 3.4.3, after placing the bids to the tasks, the auction host will calculate the comprehensive benefit and decide the task candidates. Meanwhile, the capability matrixes of each UAV will change correspondingly. When the auction for all the tasks is finished, the final capability matrixes indicate the Pareto optimal strategy for the mission. 

**Figure 18:**
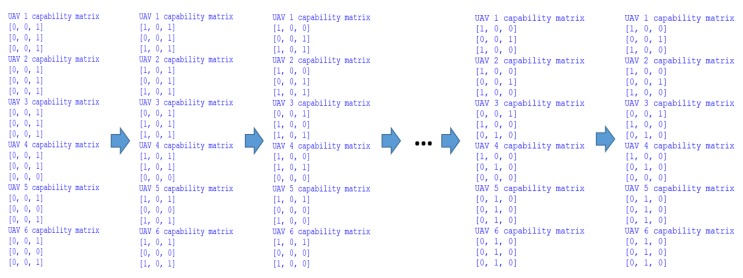
Capability matrixes changes during the negotiation process.

**Figure 19:**
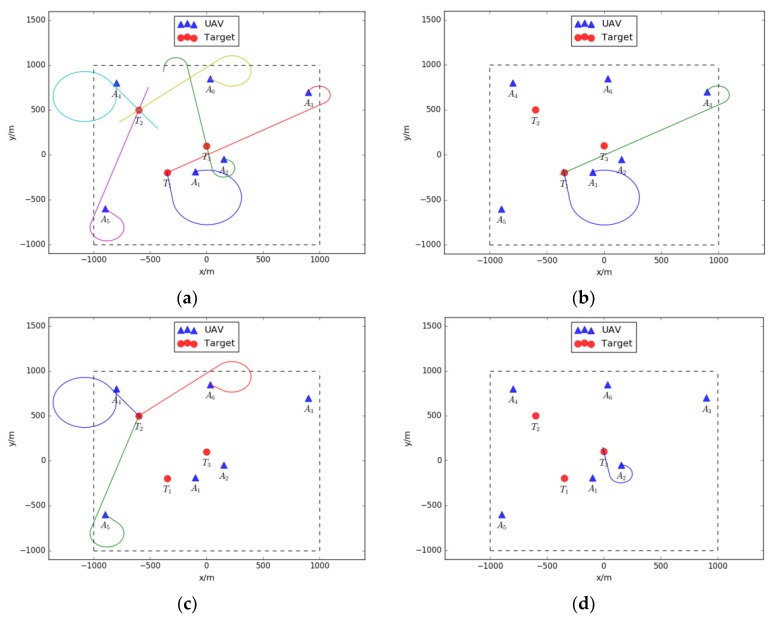
The simulation result: (**a**) overall view of the strategy; (**b**) the strategy for the first target; (**c**) the strategy for the second target; (**d**) the strategy for the third target.

In this simulation, part of the capability matrixes changes is shown in the following figure. As shown in Figure 18, the capability matrixes of each UAV change according to the negotiation results. It must be noted that the last two stages illustrate the same capability matrixes, because the Pareto optimal strategy is found at the end of negotiation process. To clearly present the generated Pareto optimal strategy for the pesticide spraying mission, the strategy is drawn in Figure 19.

**Figure 20:**
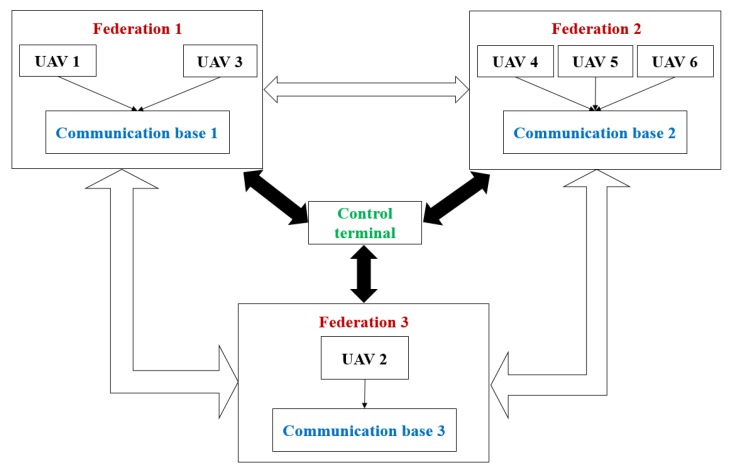
Agent formation in pesticide spraying mission.

As shown in Figure 19a–d, the first target is assigned to the agent coalition with UAV 1 and 3, the second target is assigned to the agent coalition with UAV 4, 5 and 6, and the third target is assigned to UAV 2. According to Figure 19a, the six UAVs are forming improved federal architectures mentioned in Section 2.1. The construction of proposed improved federal architectures is shown in Figure 20.

As is shown in Figure 20, the control terminal is acting as captain agent and is responsible for managing among federations, generating mission planning strategies for agricultural missions and assigning the tasks to UAVs. The communication bases are acting as sub-captain agents and are responsible for transferring the messages between UAVs and the control terminal. The UAVs are acting as normal agents in the proposed improved federal architecture and are used to execute assigned tasks. In conclusion, the construction of an improved federal architecture is successful and meets our expectations. It must be noted that this paper does not consider the detail of communication between each agents. Thus, the communication process is assumed to be fully functional. Within the range of each federation there is always a communication base, responsible for receiving and sending messages between UAVs and the control terminal. 

For the purpose of illustrating how the generated Pareto optimal dominates other alternative strategies, we selected several evaluation criteria for the comparison, including energy consumption, equipment loss and arrival time to the target. The alternative strategies are selected from the Pareto optimal strategy set. The Pareto optimal and alternative strategies are listed in Table 6.

In Table 6, two alternative strategies are presented. In alternative strategy 1, the first target is assigned to the coalition with UAV 1 and 3, the second target is assigned to the coalition with UVA 2 and 6, and the third target is assigned to UAV 4. The UAV 5 is not selected to carry on the mission. In alternative strategy 2, the first target is assigned to the coalition with UAV 3, 5 and 6, the second target is assigned to the coalition with UAV 1 and 2, and the third target is assigned to UAV 4. The evaluation for these three strategies is listed in Table 7.

As is shown in Table 7, the Pareto optimal achieves the minimum energy consumption at 646.78, the minimum equipment loss at 6.89% and the shortest arrival time to the targets at 51.61 s. These numbers illustrate that the Pareto optimal dominates the alternative strategies. 

The simulation also compares the proposed MP-PSOGA with the originals PSO and GAs. The principles of these two original algorithms are already explained in Section 3.3.1. It must be born in mind that the original PSO and GAs do not have a dynamic parameters updating mechanism. It means that the parameters of these two original algorithms are fixed values. The population size of all three algorithms is set at 40 and the iteration number is set at 50. For original PSO, the weight is set at 0.9 and learning factors are set at 2.5. For original GAs, the crossover rate is set at 0.9 and the mutation rate is set at 0.1. All three algorithms are compared under the same simulation case, which is mentioned in Section 4.1. 

**Figure 21:**
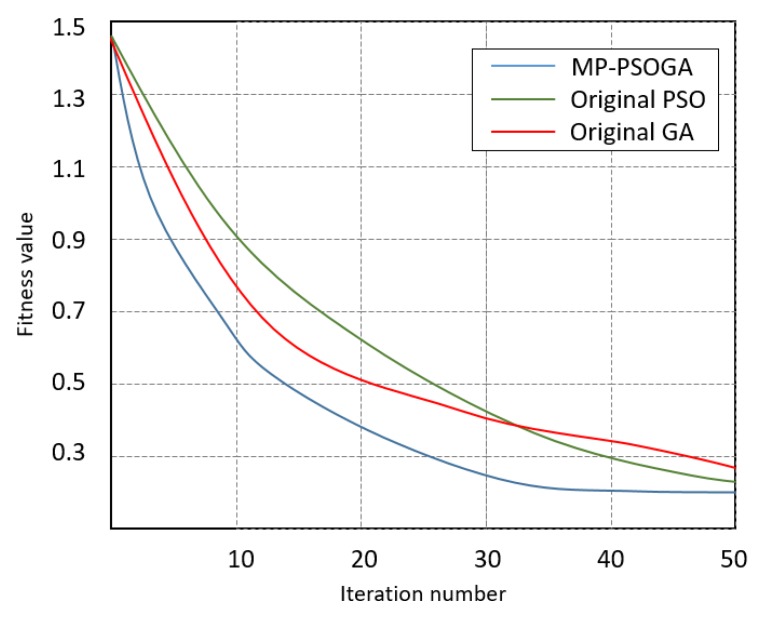
Comparison between MP-PSOGA, original PSO and GAs.

In this simulation, the changes of fitness values are compared. The lower the fitness value is, the more accurate the solution is. The comparison result is shown in Figure 21.

In Figure 21, it is clearly indicated that the fitness value of MP-PSOGA is smaller than that of the original PSO and GAs during the iteration, which means that the mission planning strategy generated by MP-PSOGA is better and more accurate. Meanwhile, Figure 21 also indicates that the convergence of MP-PSOGA is quicker than that of the original PSO and GAs, because MP-PSOGA generates the solution in a short time (the iteration number is smaller). In conclusion, the proposed mission planning approach based on MP-PSOGA dominates the original PSO and GAs. After verifying through simulations, it is concluded that the proposed mission planning approach is feasible for the precision of the farming system.

## 5. Conclusions and Future Work

The main focus of our work is on the mission planning approach for the precision farming system, along with the detailed procedures of implementation. The conclusion of our contribution and future work are summarized in this section.

### 5.1. Conclusions

Our contributions regarding the mission planning approach for the precision farming system can be described as:

- After studying the definitions and characteristics of agents and multi-agent system, the architecture of precision farming system is proposed. The precision farming system, treated as a multi-agent system, has an improved federal architecture with three types of agents, including the captain agent, sub-captain agents and normal agents. The captain agent is responsible to coordinate the whole precision farming system and establish new missions. The sub-captain agent is acting as the facilitator in the federation and is responsible to the communication. The normal agent is the task executor and has the duty of completing the assigned tasks. With such architecture, the precision farming system has the advantages of easy management, low communication cost, high intelligence, high flexibility, easy scalability and etc.
- A general target model and UAV model is proposed in this paper. Both models are implemented by the programming language Python. Relevant parameters are defined as well. Meanwhile, the functions of each model are defined in detail for the further use in the simulation. Especially in the UAV model, we considered the situation that the UAV may fly beyond the pre-defined border. So, a border-handling function is defined to calculate the minimum turning angle and generate a flying path back to the farming land.
- For the precision farming system, two objective functions are defined mathematically, including the beneficial and cost objective functions. Regarding the mission planning, it is treated as a multi-objective optimization problem, because it considers several criteria, including energy consumption, equipment loss, expected benefit and working efficiency.
- In order to generate the mission planning approach, we studied two widely-used optimization algorithms, Particle Swarm Optimization and Genetic Algorithms. We took advantage of both algorithms and developed an improved optimization algorithm, called MP-PSOGA. The main body of MP-PSOGA is based on Particle Swarm Optimization, embedded with crossover and mutation operators from Genetic Algorithms. Meanwhile, we developed dynamic updating mechanism for the parameters of MP-PSOGA, including weight, learning factors, crossover and mutation probabilities. With these dynamic parameters, MP-PSOGA is capable of computing the precise Pareto optimal with quick convergence. The simulation results illustrate that the proposed mission planning approach works properly.
- An agent coalition mechanism is proposed for the purpose of enabling the UAVs to work cooperatively in the precision farming system. The coalition formation is based on the negotiation result. During the negotiation process, a mission auction is held in order to generate the most appropriate agent coalition for a specific task. The auctioneer computes the comprehensive benefit by running MP-PSOGA. The negotiation process aims to distribute the benefit in a balanced way.
- Dynamic changes of precision farming system is taken into consideration in this paper. The dynamic changes includes establishment of new missions, malfunction of UAVs and change of meteorological environment. Hence, a mission re-planning mechanism is introduced into the precision farming system, aiming to re-assigning tasks based on new situation. The mission re-planning mechanism strengthens the robustness of the precision farming system.

### 5.2. Future Work

In this paper, most of the work for the precision farming system focus on the theoretical study and simulation verification. Though the simulation result illustrates the feasibility of the proposed mission planning approach, it is more convincible if this mission planning approach is verified in the real scenario by deploying the approach in real UAVs. However, several work and studies should be done before the deployment. The future work is summarized as follows:

1) In this paper, only a general target model and UAV model are defined. Though other agricultural machinery models, such as unmanned ground vehicles and harvesters, are similar to the UAV model, it is necessary to define these models and simulate them to verify their usability in precision farming system. Meanwhile, a management module in precision farming system should be considered in order to manage all the agricultural machineries. Regarding the target model, it is possible to implement the second development on the general target model. For example, to implement the livestock model, it is feasible to add random movement into the general target model. In future study, these theoretical models should apply to the real scenario. Fortunately, there is one development tool, called “Micro Python”, allowing us to develop the real agricultural machineries with ARM processors or field programmable gate array (FPGA) by means of the programming language Python. The syntax of Micro Python is almost the same as Python 3.* and Micro Python has its own translator, interpreter, library and etc. Hence, it is promising and feasible to test the proposed mission planning approach in the real agricultural machineries in the future. Furthermore, it is necessary to verify the proposed mission planning approach under a large-scale case. For example, thirty target fields and twenty agricultural machineries.
2) A credit evaluation mechanism should be taken into consideration. During the negotiation process, each agent is supposed to not only consider the benefit of itself, but also the benefits of other agents’. However, sometimes, certain agents are selfish and only consider their own benefits. These selfish agents may maliciously occupy the limited resources and not share information with other coalition members. Under such circumstance, other agents are unable to compete these selfish agents in the auction process and no long receive any tasks due to lacking of resources. So, the credit evaluation mechanism aims to supervising the behavior of each agent. When an agent is found to behave selfishly, then, certain punishment should be established, such as forbidding this selfish agent bidding in the next-round auction. In addition, if an agent is unable to complete the assigned task, its credit should be deducted by certain number. For these agents, who complete the assigned tasks successfully, certain credits should be rewarded. Generally, the credit evaluation mechanism ranks each agent’s reliability. A higher credit an agent has, the more reliable this agent is. Such credit evaluation mechanism could strengthen the robustness of precision farming system.
3) During the negotiation process, the auction mechanism is proposed in order to distribute the decomposed tasks to agricultural machineries. In our work, the auction mechanism is based on English Auction (EA), also known as Open Ascending Bid (OAB). However, there are several types of auction mechanisms, such as sealed-bid auction, the first-price sealed auction, Vickrey auction and etc. In the future work, these mentioned auction mechanisms could be combined in order to improve the performance of negotiation process. Furthermore, it is possible to propose more mechanisms into the negotiation process, such as Nash bargaining, cooperative game theory, non-cooperative game theory, etc. By proposing more negotiation mechanisms, the precision farming system may be more applicable to the real scenario and achieve better performance.
4) In this paper, mission re-planning process is not considered thoroughly. When dynamic changes occur, the captain agent will calculate the optimal mission planning strategy for the new mission from the very beginning, which lacks of consideration for the already-completed parts. Ideally, in the precision farming system, UAVs should have the capability of dynamically adapting to new missions without restarting from the beginning. Because re-planning from the very beginning will cost more resources and computation time than re-planning from the break point of the system. Thus, it is worthy of introducing an efficient mission re-planning mechanism in order to enhance the robustness of PFS.

## Figures and Tables

**Table 1:** Attributes of target model.

Position in the Array	Content
[0:1]	Target_position
[2]	Required_resource
[3]	Required_priority
[4]	Target_condition

**Table 2:** Attributes of UAV model.

Position in the Array	Content
[0:1]	UAV_position
[2]	Phi
[3]	Velocity
[4]	R_min
[5]	Detect_scope
[6]	Loaded_resource
[7]	Remaining_energy
[8]	Energy_consumption_rate
[9]	Equipment_Status
[10]	Equipment_loss_rate

**Table 3:** Detail of fields and required resources.

Field Number	Pesticide Type	Pesticide Quantity	Time Requirement	Position	Priority
1	*P_1_*	3	Within 1 h	(−350, −200)	3
	*P_2_*	5			
	*P_3_*	4			
2	*P_1_*	3	Within 2 h	(−600, 500)	2
	*P_2_*	1			
	*P_3_*	2			
3	*P_1_*	0	Within 1.5 h	(0, 100)	1
	*P_2_*	0			
	*P_3_*	1			

**Table 4:** Loading detail of six UAVs.

UAV Number	Pesticide Type	Pesticide Quantity	Position
1	*P_1_*	2	(−100, −190)
	*P_2_*	2	
	*P_3_*	3	
2	*P_1_*	2	(150, −150)
	*P_2_*	0	
	*P_3_*	1	
3	*P_1_*	1	(900, 700)
	*P_2_*	3	
	*P_3_*	2	
4	*P_1_*	1	(−800, 800)
	*P_2_*	2	
	*P_3_*	1	
5	*P_1_*	1	(−900, −600)
	*P_2_*	2	
	*P_3_*	0	
6	*P_1_*	1	(30, 850)
	*P_2_*	1	
	*P_3_*	3	

**Table 5:** The initial setting for parameters of MP-PSOGA.

*W*	*C*_1_	*C*_2_	*P_c_*	*P_m_*
0.9	2.5	2.5	0.9	0.1

**Table 6:** The Pareto optimal and alternative strategies.

Strategy	Target 1	Target 2	Target 3
Pareto optimal	Coalition (UAV 1 and 3)	Coalition (UAV 4, 5 and 6)	UAV 2
Alternative strategy 1	Coalition (UAV 1 and 3)	Coalition (UAV 2 and 6)	UAV 4
Alternative strategy 2	Coalition (UAV 3, 5 and 6)	Coalition (UAV 1 and 2)	UAV 4

**Table 7:** The evaluation for Pareto optimal and alternative solutions.

Strategy	Energy Consumption	Equipment Loss	Arrival Time (Second)
Pareto optimal	646.78	6.89%	51.61
Alternative strategy 1	816.70	11.58%	65.35
Alternative strategy 2	927.74	15.77%	63.47
